# Congenital urethral sphincter mechanism incompetence: observational clinical findings and treatment outcomes—a small retrospective study in 19 bitches

**DOI:** 10.1186/s13028-025-00841-6

**Published:** 2026-01-22

**Authors:** Pierre Langer, Charles Porsmoguer, Géraldine Bolen, Annick Hamaide, Stéphanie Noël

**Affiliations:** Department of Companion Animal Clinical Sciences, Faculty of Veterinary Medecine, University of Liège, Liège, Belgium

**Keywords:** Congenital urethral sphincter mechanism incompetence, Dog, Incontinence, Neutering, Urodynamics

## Abstract

**Background:**

This study aimed to report observational clinical findings and treatment outcomes in a population of bitches affected with congenital urethral sphincter mechanism incompetence (CUSMI). Response rate to sympaticomimetic drugs has not yet been reported in the literature in dogs affected with CUSMI. Juvenile bitches showing clinical signs of urinary incontinence were included. A diagnosis of CUSMI was made based on history, clinical signs, confirmation of orthotopic ureters and urethral pressure profilometry. At first consultation, all bitches (neutered or not) received phenylpropanolamine 1.5 mg/kg orally *Semel in Die* for at least one month. Median short-term (32 days) and long-term (38.7 months) follow-up data were collected via physical recheck, telephone or email questionnaire with owners, to include continence scores.

**Results:**

Nineteen bitches met the inclusion criteria. At the time of diagnosis, there was no significant difference of continence score between neutered and non-neutered bitches (*P* = 0.14). During the course of the study, 17 out of 19 bitches were neutered: nine were neutered before the first heat (before heat group) and eight after the first heat (after heat group). Two bitches were non neutered at long term follow-up. After phenylpropanolamine treatment, continence score was not significantly different between the two groups (*P* = 0.71) with a median continence score of 4 (2–5) for the before heat group and a median continence score of 4 (3–5) for the after heat group. 26% of bitches (5/19) were fully continent after phenylpropanolamine treatment at short term follow-up. Continence score after treatment (median = 4 [2–5]) was significantly higher than continence score at diagnosis (median = 2 [1–4]) (*P* < 0.001). In the after heat group, temporary improvement of continence scores was observed before or during estrus in three out of eight dogs. Worsening of continence score was reported after neutering in 5/17 dogs. At long-term follow-up, 6/19 dogs were fully continent, 5 of them receiving additional treatment.

**Conclusion:**

Based on the results of this retrospective study, CUSMI may show a low rate of initial complete response achieving full continence status after phenylpropanolamine treatment at 1.5 mg/kg SID orally when compared to treatment of acquired urethral sphincter mechanism incompetence patients. Observational clinical findings suggest that worsening of incontinence may possibly occur after neutering including dogs that experienced improvement before or during estrus.

**Supplementary Information:**

The online version contains supplementary material available at 10.1186/s13028-025-00841-6.

## Background

Urethral sphincter mechanism incompetence (USMI) is a relatively frequent condition in female dogs [1–5]. It is characterised by involuntary urine leakage which is exacerbated when the animal is asleep, recumbent or excited.

Acquired USMI (AUSMI) is most frequently observed in neutered, adult, medium to large breed bitches, with a reported prevalence of 3 to 20% [1–5].

Congenital USMI (CUSMI) is observed in juvenile dogs and the prevalence is yet unknown [6]. CUSMI has been described in medium to large breed dogs, with Springer Spaniel breed dogs being most frequently reported [7]. Although considered as the second most likely cause of congenital incontinence after ectopic ureter (EU), there is a lack of literature regarding CUSMI [7]. CUSMI can occur concurrently with congenital urogenital abnormalities such as EU, vaginal abnormalities, or hypoplastic (short) urethra [8, 9]. The type of incontinence generally differs from EU, which is mostly associated with permanent dripping of urine, whereas CUSMI is generally associated with loss of urine while the dog is asleep or when being excited [7, 9]. This clinical presentation in puppies can easily be misinterpreted with house-training accidents and may delay the diagnosis.

In cases of AUSMI, medical treatment is the first line treatment, which includes the administration of α-adrenergic agonists, gonadotropin-releasing hormone analogues, and estrogens. Alpha-adrenergic agonists are considered as the most efficient treatment with continence observed in 86 to 100% of treated dogs [10–13]. Where medical intervention fails, surgical treatment (artificial urethral sphincter placement, colposuspension) or injection of urethral bulking agents such as cross-linked gelatin (VetFoam™) may also be considered [14, 15]. In case of CUSMI, only estrogen treatment has been previously described with no reported outcome [7].

Up to 50% of bitches with CUSMI may show continence improvement after the first or second estrus [16, 17]. In female Beagle dogs, improvement of the urethral sphincter function has been reported by carrying out urodynamic examinations during the prepubertal period. Urethral resistance increased when dogs were older than 7 months old, as well as after the first and second estrus [18].

To the authors’ knowledge, treatment outcomes in dogs affected with CUSMI and treated with a first line α-adrenergic drug have not yet been reported. The objectives of this study are twofold: firstly, to report observational clinical findings in a population of nineteen bitches affected with CUSMI in order to further document this condition and, secondly, to report the outcome in those bitches treated with an initial administration of phenylpropanolamine (PPA).

## Methods

### Inclusion criteria

Medical records of bitches presented to our university referral hospital for urinary incontinence referral between January 2015 and April 2024 were retrospectively reviewed. Data included breed, age, neuter status, time of neutering (before the first heat (BH) or after the first heat (AH)), diagnostic imaging findings, urodynamic findings, final diagnosis and treatment. Ultrasonography was the first line imaging modality. Variations in the choice of additional imaging modalities were made based on clinician’s decision at the time of first consultation, as well as owners’ choice regarding anesthesia and costs.

At first consultation, discharge instructions were made for a recheck between 3 and 6 weeks. Short term follow-up post PPA treatment was obtained by anamnesis and recorded by two of the co-authors either at our university referral hospital or by telephone or e-mail. Standard questions in order to assess continence score (amount, frequency, pattern of micturition) were asked by both clinicians. Long-term outcome was determined based on owners’ questionnaire via telephone/e-mail.

Bitches included in the study had signs of urinary incontinence which were consistent with clinical signs of USMI such as worsening of urine leakage during excitement, while sleeping or being recumbent, based on the anamnesis from the owners and the medical records from the referring veterinary surgeon. Those signs were reported during their juvenile period and prior to neutering. Presence of EU, vaginal abnormalities as well as hypoplastic (short) urethra was ruled out.

### Diagnostic procedures

A complete blood analysis (hematology and biochemistry), urinalysis (urine dipstick and urine cytology), as well as urine culture (if infection was suspected) were performed before the diagnostic imaging procedures to exclude urinary tract infection or renal disease.

Diagnostic imaging techniques included abdominal ultrasonography (US), abdominal radiographs following intravenous (IV) pyelogram combined with pneumocystography and retrograde vagino-urethrography using fluoroscopy to dynamically assess the ureteral jets, computed tomography (CT) with excretory urography, as well as cystoscopy. During abdominal ultrasonography, furosemide (Dimazon™, Intervet, Belgium) was administered IV (0.3 mg/kg) to improve the visualization of the ureteral jets into the bladder.

After exclusion of the presence of EU, vaginal abnormalities and hypoplastic (short) urethra, urodynamic examination was performed under a light and stable plane of anesthesia obtained with an IV continuous rate of 1 mg/kg/min of propofol (Propovet™, Zoetis, Belgium) [19]. Urethral pressure profilometry (UPP) and retrograde filling cystometry were performed as previously described [19]. Detailed description of UPP parameters can be found in additional file 1 [20]. Medications with urinary incontinence tropism administered by the referring veterinarian were discontinued at least one week prior to consultation in order not to interfere with urodynamic results and interpretation.

### Data analysis

A continence score (CS) was defined and rated out of five as previously described with a score of 1 representing a fully incontinent bitch and a score of 5 representing complete continence [21] (Table 1).

**Table 1:** Continence score (CS) as described by Byron et al., 2007

Score	Description
1	Dog is never continent. Dribbles urine when awakes as well as when sleeping. Constantly has a wet perineum and leaves urine on surfaces when getting up from a sitting or recumbent position.
2	Poorly continent. Dog urine soils where it has been sleeping more than 50% of the time. Dribbles urine or has a wet perineum when awake 25–75% of the time.
3	Dog urine soils where it has been sleeping more than 50% of the time. Dribbles urine or has a wet perineum awake, up to 25% of the time.
4	Dog urine soils where it has been sleeping up to 50% of the time, but does not dribble urine or have a wet perineum when awake.
5	Dog is always continent.

The CS of all bitches was determined at the time of diagnosis of CUSMI (first consultation at our hospital), at the short-term (approximately one month after initiation of treatment) and at long-term (telephone or e-mail interview) evaluations. For intact bitches, the CS was also recorded around their heat period.

The interview consisted of a standardised questionnaire (see additional file 2) on signs of urinary continence before and after treatment, current medications and potential episodes of urinary tract infection, as well as around heat period. The CS was explained and assessed with the owner.

Normality assessment was performed with the Shapiro-Wilk test and with graphical representation. As the distribution of all variables were non normal, descriptive data were reported as median and range. Statistical comparison of CS was performed using a paired Wilcoxon test to compare CS before and after PPA treatment and to compare CS pre- and post-neutering. A Mann-Whitney test was used to compare CS at the time of first consultation between neutered and non-neutered groups, to compare CS of the BH and AH groups after PPA treatment and to compare CS after neutering in different groups (BH vs. AH). A P value < 0.05 was considered statistically significant. Statistical analysis was performed using RStudio (R package version 4.4.1) (R: A language and Environment for Statistical Computing_.© R Foundation for Statistical Computing, Vienna, Austria).

## Results

Nineteen bitches met the inclusion criteria. The median age at the first consultation was 12 months (4–72). All dogs presented with signs of urinary incontinence during their juvenile period and before neutering. One bitch was a Maltese and 18/19 dogs were medium to large breed (Table 2).

**Table 2:** Characteristics of bitches and evolution of continence score

	Breed	Age at first consultation (months)	Neutering before first heat	Continence score at first consultation at our university hospital/ diagnosed CUSMI (/5)	Continence score at short-term follow-up (/5)	Continence score at long term follow-up (/5)
1	Maltese	54	Yes	1	4	5
2	Giant Schnauzer	36	No	2	5	5
3	Dalmatian	6	Yes	1	4	4
4	Mixed breed	36	Yes	2	4	4
5	White Swiss Shepherd	12	No	2	3	3
6	Greater Swiss Mountain dog	30	Yes	1	3	4
7	Newfoundland	12	Yes	1	2	2
8	American Staffordshire Terrier	4	No	3	3	5
9	Golden Retriever	12	Yes	1	5	5
10	Border Collie	7	Yes	2	4	3
11	Border Collie	10	No	3	5	4
12	Doberman	12	No	4	4	4
13	Giant Schnauzer	72	Yes	2	3	3
14	Weimaraner	8	N/A	2	3	4
15	American Staffordshire Terrier	9	Yes	3	5	5
16	French Bulldog	8	No	2	4	4
17	Border Collie	7	No	3	4	4
18	Bullmastiff	24	No	3	4	3
19	Greater Swiss Mountain dog	10	N/A	2	5	5
Median		12		2	4	4

N/A: not applicable as non-neutered during the course of the study

CUSMI: congenital urethral sphincter mechanism incompetence

At the time of diagnosis, nine bitches were neutered (CS = 2, 1–3) and 10 bitches were intact (CS = 2, 1–4; *P* = 0.14). Prior to referral, six bitches had received PPA (Propalin™, Vétoquinol, Belgium) at 1 mg/kg *Per Os*,* Ter In Die (PO*,* TID)*, three bitches had received PPA associated with estriol (Incurin™, MSD, France) 0.5-1 mg PO *Semel In Die* (SID), one bitch had received estriol 1 mg PO SID. Other treatments included antibiotics to treat urinary tract infection (6 bitches), non-steroidal anti-inflammatory drug (NSAID) associated with antibiotics (one bitch) and NSAID alone (one bitch).

Regarding the time of neutering during the course of the study, nine bitches were neutered before the first heat (BH group) and eight after the first heat (AH group; Table 2). Two bitches were non neutered at the time of long-term telephone/e-mail interview follow-up. Among the seventeen bitches neutered during the course of the study, seven were neutered before PPA treatment and 10 were neutered after the onset of PPA treatment. The median long-term follow-up time was 38.7 (8–81) months.

A complete blood analysis (biochemistry and hematology) was available for 13 dogs. Nine were within normal limits. One dog had a mild increase in Alkaline Phosphatase (ALP) (87UI/L > 74UI/L), one dog had mild hyperkalemia (6.1mmol/L > 5.5mmol/L), one dog had mild anemia (33.5%<37.3%) and one dog had mild increase in creatinine (171 > 159 µmol/L). Nine out of 19 bitches had their urinalysis performed at the referring veterinarian. Six out of those nine bitches had urine culture performed which were all positive. Seven out of those nine bitches had episodes of urinary tract infection (UTI) treated medically after urine culture by the referring veterinarian before the referral consultation (two with amoxicillin-clavulanate (Clavaseptin™, Vetoquinol, Belgium) 12.5 mg/kg PO *Bis In Die* (BID) for 14 days, two with cefalexin (Rilexine™, Virbac, Belgium) 15 mg/kg PO BID for 10 days and three bitches were treated for 10 days with antibiotics but the drug was not specified and urine culture was not performed for one dog). Two out of those nine bitches had their urinalysis within normal limits. On the day of consultation at our university referral hospital, besides urinary incontinence, none of those nine bitches showed clinical signs of UTI such as hematuria or pollakiuria. The ten other bitches had their urinalysis performed by cystocentesis at our university referral hospital, all urine sediments were negative for bacteria and cells at cytology.

Data regarding imaging procedures are detailed in Table 3. Eighteen out of nineteen dogs had an abdominal ultrasound performed. No significant abnormality was found on abdominal ultrasonography. Each dog had two different ureteral jets that were visualised in the bladder after furosemide injection, demonstrating an orthotopic position of the ureters. Intravenous urography followed by vagino-urethrography was performed in 9/19 dogs and 8 other dogs had a vagino-urethrography without IV urography, confirming normal positioning of the ureters and revealing a mild intrapelvic bladder in four bitches, a piriform bladder with a relatively large urethra in one dog, a possible urethral flange in one dog and a possible vaginal flange in one dog. Results of intravenous urography combined to vagino-urethrography or by vagino-urethrography alone were normal in 10/17 dogs. Three dogs out of 19 had a CT excretory urography and 4/19 dogs had a cystoscopy performed, which confirmed orthotopic positioning of the ureters. All diagnostic imaging procedures were performed by ECVDI specialists.

**Table 3:** Modalities of diagnostic imaging

	Abdominal US	IV Urography	Vagino-urethrography	CT scan	Cystoscopy
Dog 1	•	•	•	–	–
Dog 2	•	•	•	–	–
Dog 3	•	•	•	–	–
Dog 4	•	•	•	–	–
Dog 5	–	•	•	•	–
Dog 6	•	•	•	–	–
Dog 7	•	•	•	•	•
Dog 8	•	–	–	–	–
Dog 9	•	•	•	–	•
Dog 10	•	–	•	–	–
Dog 11	•	•	•	•	–
Dog 12	•	–	•	–	–
Dog 13	•	–	•	–	–
Dog 14	•	–	•	–	–
Dog 15	•	–	•	–	–
Dog 16	•	–	•	–	–
Dog 17	•	–	•	–	–
Dog 18	•	–	•	–	•
Dog 19	•	–	–	–	•

• : exam performed IV urography: intravenous urography

–: exam not performed

Abdo US: abdominal ultrasound CT scan: computed tomography

Once initial treatments from the referring veterinarians were discontinued, UPPs were performed in 18 dogs and were consistent with USMI (Table 4). One dog did not have UPPs performed as the owners declined this aspect of investigation. In 10 dogs, following imaging diagnostics and UPP results, cystometry was not performed if not indicated. Due to the long-time span of the study (ten years), our standards regarding urodynamic examinations evolved over time and progressively did not involve cystometry if no signs of spastic bladder were present upon imaging diagnostics.

**Table 4:** Results of urethral pressure profilometry and cystometry

	MUP (cmH2O)	MUCP (cmH2O)	FPL (mm)	IP (cm.cmH20)	TV (ml)	TP (cmH2O)	C
Dog 1	21.5	17.5	44.5	49	*	*	*
Dog 2	22	22	68	76	487	32	18.7
Dog 3	10	12	65	38	#	#	#
Dog 4	24.3	21.5	64	53.5	292	28	12.7
Dog 5	33	29	62	84	498	43	14.6
Dog 6	29	28	68	55	352	20	22
Dog 7	10	7	45	28	#	#	#
Dog 9	35.5	33	52	80	284	14	21.8
Dog 10	25	19.3	62.3	88	159	23	10.6
Dog 11	52	50	50.5	122.5	222	29	12.3
Dog 12	80	77	89	293.5	#	#	#
Dog 13	5.5	5.5	73.5	+	#	#	#
Dog 14	6	20	25	13	#	#	#
Dog 15	32.5	30.5	72	100.5	#	#	#
Dog 16	10	7	38	30	#	#	#
Dog 17	19.3	17.6	55.6	61.3	154	8	19.2
Dog 18	18	12	55	62	#	#	#
Dog 19	26.5	24.5	132	136.5	#	#	#
Mean	24.1 (SD 17.3)	25.2 (SD 17.7)	45.5 (SD 26.4)	77.2 (SD 61.1)	305.8 (SD 124.5)	24.6 (SD 10.2)	15.2 (SD 4.3)

MUP: maximum urethral pressure

MUCP: maximum urethral closure pressure

FPL: functional profile length

IP: integrated pressure

Vth: threshold volume

Pth: threshold pressure

C: bladder compliance

*Fluid leakage at the start of bladder filling impeding data recording

# Cystometry not performed

+ Non measurable

At the time of diagnosis and before initiation of treatment at our institution, the nine bitches already neutered had a CS = 2 (1–3) and the nine non-neutered bitches had a CS = 2 (1–4) with no significant difference of CS between those two groups (*P* = 0.14).

After the diagnosis of CUSMI and exclusion of the presence of EU, all bitches received PPA at 1.5 mg/kg PO SID for at least one month. Short term follow-up post PPA treatment occurred at our university referral hospital (2 dogs) or by telephone or e-mail (17 dogs). The two dogs rechecked at our university hospital had urinalysis performed and urine sediments were negative for bacteria and cells at cytology. The median short-term recheck time was 32 days (20–158).

At short term follow up, CS was significantly higher than before treatment (*P* < 0.001). The median CS for all bitches at the time of diagnosis was 2 (1–4) whereas the median CS after PPA treatment was 4 (2–5). There was no significant difference in CS after treatment between AH and BH groups (*P* = 0.71) with a median CS = 4 (3–5) for the AH group and a median CS = 4 (2–5) for the BH group (Table 2).

Only 5 bitches (26%) were fully continent after initial PPA treatment of at least one month. Although not being fully continent, another 47% (9/19) of bitches had a CS of 4 (Table 2).

In the group of eight bitches which were neutered after the first heat, improvement of clinical signs occurred before or during estrus for three of them, and data collection was missing for one of them. Improvement occurred two months before the first heat for one bitch and during the first estrus for the two other bitches. Two of those four dogs remained fully continent during two and a half and four months respectively, but clinical signs relapsed after neutering.

Overall, a significant decrease in CS was found after neutering (*P* = 0.047). Worsening of incontinence after neutering was observed in five dogs (31%) (two out of the nine bitches of the BH group and three out of the eight dogs of the AH group), data collection was missing for one of them (Table 2). The difference of CS after neutering between AH and BH groups was not significant (*P* = 0.62) with a median CS = 4 (2–5) for the AH group and a median CS = 3 (1–5) for the BH group.

In total, twelve bitches had changes in their medical treatment during the long-term follow-up period. Treatment was adapted in these dogs as they were not responding well to the initial PPA dosage. UTI was ruled out at the referring veterinarian before implementing the treatment changes.

The dose of PPA was modified in eight bitches (PPA 1.5 mg/kg BID (6 dogs); PPA 1.5 mg/kg every other day (two dogs)) and the treatment molecule was switched to ephedrine 1 mg/kg BID (Enurace™, Virbac, Belgium) in four bitches after worsening of CS during long term treatment with PPA. Regarding the 6 bitches receiving 1.5 mg/kg of PPA BID and 3 out for 4 that received ephedrine instead of PPA, the new therapeutic plan allowed improvement of the continence and return to the CS recorded at the start of the initial treatment of PPA 1.5 mg/kg SID. The bitches that received PPA every other day were fully continent before the new dosage was implemented and remained fully continent thereafter. Colposuspension was performed in two bitches to improve continence (one had a mild pelvic bladder): two polypropylene (Prolene™) sutures (size 2 − 0) were placed on each side of the urethra.

In one bitch, colposuspension was performed during neutering, 9 months after initiation of medical treatment, and the bitch showed a worse CS after the procedure (from 4 to 3). This bitch had adaptation of the long term PPA regimen (PPA 1.5 mg/kg BID). In the other bitch, CS improved from two to three after colposuspension performed six months later after initiation of medical treatment and she had no adaptation of the PPA regime.

At long term follow-up interview, 6/19 bitches (31%) were fully continent: 4/19 (21%) with medical treatment only, 1/19 without medical nor surgical treatment (5%) and 1/19 (5%) with a combined medical and surgical treatment. 73% (14/19) of the bitches had a CS higher or equal at 4 out of 5. The second dog that had a colposuspension procedure was on PPA 1.5 mg/kg BID. Regarding the bitches that were not fully continent and did not have a colposuspension procedure, three were on PPA 1.5 mg/kg BID, one was on PPA 1.5 mg/kg every other day, two were on PPA 1.5 mg/kg SID, one was on ephedrine 1.5 mg/kg SID, three were on ephedrine 1 mg/kg BID and two bitches were not on any treatment.

## Discussion

In this retrospective study, improvement of continence before or during estrus was observed as well as worsening of incontinence after neutering in some bitches. Response of CUSMI condition to the initial medical treatment was low with only 26% of bitches being fully continent after approximately one month of PPA treatment at 1.5 mg/kg SID. At long-term follow-up, after treatment adjustments, the continence was improved in all bitches, with 31% of the bitches being fully continent and a total of 73% of bitches having a CS equal or superior to 4.

Improvement of urinary incontinence may occur before or during the first estrus [7]. In our study, improvement of continence before or during estrus occurred in 3 out of 7 bitches with two being fully continent although those two bitches became incontinent again after neutering. Those findings are consistent with the existing literature which reports about half of the bitches becoming fully continent after the first or second estrus [7]. Based on those observational findings, one might suggest for future recommendations not to neuter bitches diagnosed with CUSMI before the first or second estrus. However, further prospective studies on a larger population would be warranted to support the potential role of neutering in bitches diagnosed with CUSMI. In one of those three dogs who had an improvement of the CS, improvement occurred at 7 months of age, which was two months before the first estrus. This may support a role of puberty or growth in the improvement of urinary incontinence. An increase in urethral resistance has been described around 7 months of age in prepubertal female Beagle dogs [18]. Urethral lengths were also increased during prepubertal period and attributed to growth in females. Increase of urethral lengths and pressures were reported until the second estrous cycle in female Beagle dogs. In young littermate female Beagle dogs, all 5 bitches had intermittent intrapelvic bladder during the prepubertal period without incontinence [18]. Improvement of incontinence before or during estrus in dogs with CUSMI could therefore be attributed to growth and puberty or could be explained by a spontaneous resolution of an intrapelvic bladder condition after the first estrus. In our study, two dogs showed a mild pelvic bladder at presentation and before the first estrus; one of the two dogs had her continence improved during puberty. Vagino-urethrography was not repeated in those two bitches which were neutered before their first heat, therefore spontaneous resolution of intrapelvic bladder condition could not be verified.

The decrease in urethral tone in bitches affected with AUSMI has been described with the development of urodynamic techniques [22]. Significant decrease in MUP, MUCP and FPL is observed in dogs suffering of AUSMI compared to continent dogs [22].

In a previous study, Noël and collaborators investigated the urodynamics values, with the same anesthetic conditions as in this present study, in normal intact prepubertal and young adult Beagle bitches and recorded a mean MUCP value of 62 cmH_2_O at 9 months (before the first heat), 70 cmH_2_O during their late anestrus phase of their first estrus cycle and a mean MUCP value of 80 cmH_2_O during their late anestrus phase of their second estrus cycle [18]. In the same study, the threshold volume increased at 6 months and remained stable until the end of the prepubertal period, while the bladder pressure increased from 7 to 9 months. On the other hand, bladder capacity and compliance were similar between the end of the prepubertal period and the second anoestrus, suggesting that, in dogs, except during the periods with sexual hormonal impregnation, the bladder capacity is reached at the end of the prepubertal period [18]. At the time of first consultation, the age of most of the bitches included in the present study was comparable to the age of the continent Beagle dogs used in Noël’s previous study. In our study, the mean MUCP value was 24.1 cmH_2_O.This lower mean MUCP value may further reflect the CUSMI condition of our bitches. In the present study, the results of the cystometries were unremarkable. Further prospective studies measuring UPP values in a larger number of bitches might be warranted to assess the reliability of this modality as a complementary diagnostic tool for CUSMI investigation.

Despite Springer Spaniels being the most reported breed with CUSMI, we did not encounter this breed in our population, potentially related to the geographic location and inhabitant’s breed preference where our study was conducted.

In our study, despite improvement of continence reported before or during the first estrus in 3 out of 7 bitches, worsening of incontinence was reported by the owner of one of those bitches. Functional modifications of the lower urinary tract with variations in circulating hormones during estrous cycle have been described [18, 23]. The two concomitant hormonal changes (estrogens and progesterone) may lead to a reduced urethral tone as reported in a previous study [23] and explain the worsening of incontinence in our fourth bitch.

While AUSMI is reported in the literature in 3 to 20% of neutered bitches which are initially not affected with CUSMI [1–5], we observed in the present study a worsening of incontinence after neutering in 5 out of 16 dogs (31%) with CUSMI. To date, there is no known hypothesis to support this finding. In authors’ opinion, incontinence after neutering in CUSMI dogs may worsen as there is a reported decreased urethral closure pressure within 1 year after neutering [24] which, in turn, may further participate to incontinence on an already weak urethral sphincter in CUSMI bitches.

In our study, all bitches received the same initial treatment consisting of administration of PPA at 1.5 mg/kg SID solely. Only 26% of bitches were fully continent after this initial treatment. Therefore, the medical treatment had to be adapted. Six bitches had modification of their PPA regimen from once to twice a day, which led to an improvement of CS. Prolonged administration of α-adrenergic drugs may cause a right shift in the dose-response curve hence reducing the maximal response to the drug [25]. This may explain clinical improvement witnessed following administration of higher daily dose of PPA. As ephedrine has been reported to be less effective than PPA in the treatment of acquired USMI, with a success rate ranging from 25 to 75% and associated with more side effects [21, 26], this molecule is usually chosen as a second line treatment after failure of PPA treatment and was used in two bitches in our study.

Estriol was not used in our study and is contra-indicated in bitches younger than one year [27]. Continence rates of 65 to 83% are reported with estriol alone for the treatment of acquired USMI [28, 29]. Furthermore, the association of estriol and PPA did not appear to be superior to administration of PPA alone in increasing urethral pressure in healthy adult Beagle dogs [30]. GnRH analog or GnRH vaccination could also have been used and may result in improvement of incontinence in dogs with acquired USMI [31, 32]. Response rate to sympaticomimetic drugs had not been reported yet in the literature in case of dogs affected with CUSMI. In bitches with AUSMI treated with various dosages of PPA, continence may be achieved in 86 to 97% of dogs [10–13]. Initial dosage of PPA at 1.5 mg/kg SID was elected by authors based on previous experimental and clinical studies showing that a BID or TID administration of PPA at 1.5 mg/kg did not significantly increase urethral resistance above baseline values and above SID administration in dogs affected with USMI [25, 33]. In a previous study including bitches with AUSMI treated with the same initial dosage of PPA as we used in the present paper, a success rate of 88% of complete continence was reported [34]. Results of the present study might suggest that dogs with CUSMI may have low response rate to initial PPA treatment with only 26% of complete continence.

Adjustment of the treatment including the use of surgical procedure during the follow-up period prevented a long-term comparison. Two dogs underwent colposuspension to improve their continence. In one of those dogs, CS improved after the procedure while CS was worse for the other dog. Colposuspension is a relatively effective procedure to improve continence in bitches with AUSMI becoming refractory to medical treatment. In a study on 150 dogs affected with AUSMI, 53% were fully continent after surgery and an improvement of continence was reported in another 37% of bitches [35]. When colposuspension is combined with urethropexy, 70% of bitches were fully continent [15, 36]. Other techniques such as artificial urethral sphincter or bulking agents could be interesting in refractory patients as they both have been described to improve continence [37–40].

73% of the bitches had a CS higher or equal at 4 out of 5 with owners reporting overall clinical improvement hence a better quality of life for the bitches. One could argue that the low success rate of the various treatments achieving full continence in the present study might be related to a potential misdiagnosed EU condition. Cystoscopy or CT excretory urography were not systematically performed in this study to exclude EU and were carried out in 4/19 and 3/19 dogs respectively. Even though cystoscopy is considered to be the gold standard technique to detect EU with a reported 100% sensitivity whilst being an operator-dependent technique [41–43], US and CT are nonetheless sensitive diagnostic imaging modalities for EU screening [9, 42, 44], although a recent study showed that CT excretory urography prediction of the location of the ureteral orifice shows low sensitivity especially in or close to the urethral sphincter area [45].

Variations in the choice of imaging modalities were made based on clinician’s decision at the time of first consultation, owners’ choice regarding anesthesia and costs which precluded additional CT scan and/or cystoscopy performed in all cases.

In our study, 18/19 dogs underwent abdominal ultrasonography performed by an experienced ultrasonographer. In a recent study, ultrasonography had a reported sensitivity of 93.5% and a specificity of 100% for the detection of EU in incontinent dogs [46]. As all the dogs screened through ultrasound examination were negative for EU, the 100% specificity inherent to the US modality and proper visualization of the two different ureteral jets in the urinary bladder after furosemide injection render the fact that it is highly unlikely that the diagnosis of EU was missed. In addition to ultrasonography, most dogs (8/19) had IV excretory urography combined with vagino-urethrography under fluoroscopy control to assess the two different ureteral jets that showed no signs of the presence of EU. Duplicate ureter with ectopia in dog has been described but is quite seldom with only two patients reported in the literature [47, 48]. It is possible that one of these particular types of EU may not have been completely ruled out although the odds are quite low given the fact there was no accumulation of contrast product caudally to the bladder after our IV urography procedures.

Limitations of this retrospective study include the small number of cases, bias and loss of information inherent to its retrospective nature as well as the presence of individual adjustments in treatment protocols during the long-term follow-up. Clinical signs attributable to CUSMI were based on the anamnesis from the owners and the medical records from the referring veterinary surgeon hence leading to potential recall bias. Information retrieved from owners regarding CS at the time of first consultation and in the long-term follow-up are subject to recall bias which may have hampered recognition of subtle details necessary for accurate CS grading. The use of Byron system with a grading between 1 and 5 may have precluded perception of more refined clinical details, potentially altering the changes of continence scores during the course of the study and underestimating the severity of each of the grades hence the quality of life of the patients. Due to the retrospective nature of the study, there is a risk of misdiagnosed UTI at the time of first consultation as all the urine samples were not collected by cystocentesis and a culture and sensitivity test was not systematically performed.

Finally, considering a future prospective study, a standardised imaging protocol would preclude the lack of repeatability in our current study.

However, as stated above, the primary goal of the study was mainly to further document the CUSMI condition in a view to contribute to a better understanding on treatment clinical responses in affected bitches. Long-term improvement or deterioration of CS was only assessed by the owner through a telephone or e-mail interview and no urodynamic examination follow-up was performed which would have allowed quantification of functional vesico-urethral changes. However, the need for general anesthesia for those procedures may preclude repetitive examinations in clinical patients.

## Conclusions

This study is the first study to describe the potentially low clinical response to a first line standardised initial treatment with PPA at 1.5 mg/kg SID in bitches with CUSMI when compared to treatment of AUSMI patients, achieving full continence status in 26% of the dogs, despite continence improvement in most cases.

Based on our observational clinical findings in our study, owners of bitches diagnosed with CUSMI may be informed of the potential risk of worsening of incontinence after the neutering procedure. Further prospective studies including a larger number of bitches would be warranted to support these results.

## Supplementary Information

Supplementary material 1. UPP variables description: notes explaining UPP parameters used in the study

Supplementary material 2. Questionnaire congenital USMI study: questionnaire filled by the owners regarding urinary behavior and continence score of their dogs

## Data Availability

The data sets used and/or analysed during the current study are available from the corresponding author on reasonable request.

## Abbreviations

AH: After first heat
ALP: Alkaline Phosphatase
AUSMI: Acquired urethral sphincter mechanism incompetence
BH: Before first heat
BID: Bis In Die
CS: Continence score
CT: Computed tomography
CUSMI: Congenital urethral sphincter mechanism incompetence
EU: Ectopic ureter
FPL: Functional profile length
IV: Intravenous
MUCP: Maximal urethral closure pressure
MUP: Maximal urethral pressure
NSAID: Non steroidal anti inflammatory drug
PO: Per Os
PPA: Phenylpropanolamine
SID: Semel In Die
TID: Ter In Die
UPP: Urethral pressure profilometry
US: Ultrasound
USMI: Urethral sphincter mechanism incompetence
UTI: Urinary tract infection

## Declarations

### Prior publication

Data have not been published previously.
