# Supplementary material for: Congenital urethral sphincter mechanism incompetence: observational clinical findings and treatment outcomes—a small retrospective study in 19 bitches
Source: Acta Vet Scand. 2026 Jan 22;68:5. doi: 10.1186/s13028-025-00841-6 (PMC12829239; doi:10.1186/s13028-025-00841-6)
Supplement: Supplementary file 1 — Supplementary material 1. UPP variables description: notes explaining UPP parameters used in the study [file 13028_2025_841_MOESM1_ESM.docx]

Additional file 1: notes explaining UPP parameters used in the study

UPP variables description

Specific variables were assessed such as maximum urethral pressure (MUP), maximum urethral closure pressure (MUCP ie, the difference between MUP and bladder pressure), integrated pressure (IP ie, the functional area under the curve), functional profile length (FPL, defined as the distance between the point in the urethra where intraurethral pressure exceeds bladder pressure and the point where either a pressure plateau is detected or where the intraurethral pressure is less than bladder pressure). Threshold pressure (Pth ie, urinary bladder pressure at the time of micturition) was also measured as well as threshold volume (Vth ie, fluid volume retrieved from the urinary bladder at the time of micturition reflex), and bladder compliance (C= (Vth-V0/Pth-P0) where V0 and P0 are the urinary bladder volume and pressure at the start of cystometric evaluation [20].
