# Supplementary material for: Congenital urethral sphincter mechanism incompetence: observational clinical findings and treatment outcomes—a small retrospective study in 19 bitches
Source: Acta Vet Scand. 2026 Jan 22;68:5. doi: 10.1186/s13028-025-00841-6 (PMC12829239; doi:10.1186/s13028-025-00841-6)
Supplement: Supplementary file 2 — Supplementary material 2. Questionnaire congenital USMI study: questionnaire filled by the owners regarding urinary behavior and continence score of their dogs [file 13028_2025_841_MOESM2_ESM.docx]

Additional file 2: questionnaire filled by the owners regarding urinary behavior and continence score of their dogs

**Questionnaire congenital USMI study :**

Name :

Breed: Sex: Sterilised: Yes / No Date of sterilisation:

Date of birth: Weight:

**Clinical signs at time of diagnosis :**

**Treatment received prior to CVU consultation :**

Yes: Which one? Improvement (continence score)?

No

**Date of diagnosis (age at dx) :**

**Continence score before treatment/at diagnosis:** (/5 Byron *et al*, 2007 JVIM)

**Treatment received after consultation (type, dose) :**

Yes, Which one?

No

**Continence score after medical treatment**: /5

**Effect of estrus on incontinence :**

Resolution? Improvement (before/during/after?)

Continence score :

Temporary?

Permanent?

**Role of neutering :**

Post-sterilisation continence score (improvement, stability or deterioration): /5

Effectiveness of post sterilisation treatment:

**Current medical treatment?**

**Recurrence/degradation of incontinence** (score /5)

Post sterilisation? If yes, for how long?

Post estrus? If yes how long?
